# Effect of Levosimendan Use on All-Cause Mortality in Out-of-Hospital Cardiac Arrest Survivors After Extracorporeal Cardiopulmonary Resuscitation

**DOI:** 10.3390/biomedicines13040955

**Published:** 2025-04-13

**Authors:** Da-Long Chen, Yu-Kai Lin, Guei-Jane Wang, Kuan-Cheng Chang

**Affiliations:** 1 Graduate Institute of Clinical Medical Science, China Medical University, Taichung 40402, Taiwan; jennyw355@gmail.com; 2 Division of Cardiovascular Medicine, Department of Medicine, China Medical University Hospital, Taichung 40447, Taiwan; mbestdsuyun@gmail.com; 3 Graduate Institute of Biomedical Sciences, China Medical University, Taichung 40402, Taiwan; 4 Department of Medical Research, China Medical University Hospital, Taichung 40447, Taiwan; 5 Pharmacy Department, Wizcare Medical Corporation Aggregate, Taichung 40404, Taiwan; 6 School of Medicine, Weifang University of Science and Technology, Weifang 262700, China; 7 School of Medicine, College of Medicine, China Medical University, Taichung 40402, Taiwan

**Keywords:** out-of-hospital cardiac arrest, extracorporeal cardiopulmonary resuscitation, levosimendan inodilator, extracorporeal membrane oxygenation

## Abstract

**Background:** Survivors of out-of-hospital cardiac arrest (OHCA) after external cardiopulmonary resuscitation (ECPR) have a mortality rate as high as 50–70%. The use of vasoactive inotropes worsen the mortality rate at admission. The administration of levosimendan within 72 h of ECPR facilitates extracorporeal membrane oxygenation (ECMO) weaning, so it is important to determine whether levosimendan improves mortality. **Methods:** This retrospective cohort study included 158 patients with OHCA of cardiac origin who had undergone ECPR and were hospitalized between January 2015 and December 2024. This study was conducted in the intensive care unit of China Medical University Hospital, Taichung, Taiwan. Twenty-three patients received levosimendan within 72 h, whereas the others did not receive levosimendan. Primary endpoints included ECMO weaning failure rate and 90-day all-cause mortality rate. Kaplan–Meier survival curve analysis was also performed. Covariates for all-cause mortality were estimated and adjusted by using Cox regression modeling. **Results:** The levosimendan group exhibited lower rates of ECMO weaning failure and 90-day all-cause mortality than the control group (13.0% vs. 52.6% and 17.4% vs. 57.0%, respectively; both *p* < 0.001). The 90-day survival curve analysis revealed that the levosimendan and control groups had survival rates of 82.6% and 43.0%, respectively (log-rank *p* < 0.001). Administration of levosimendan within 72 h resulted in a odds ratio of 0.36 (95% confidence interval: 0.18−0.79, *p* = 0.01). **Conclusions:** Administering levosimendan within 72 h of ECPR could be a protective factor in improving all-cause mortality.

## 1. Introduction

Out-of-hospital cardiac arrest (OHCA) is estimated to occur at a rate of 50–100 per 100,000 person-years worldwide [1]. Notably, one quarter of the hospitalized patients with cardiac arrests of cardiac origin receive temporary mechanical circulatory support, with 70% of patients experiencing acute myocardial infarction [2].

Initial cardiopulmonary resuscitation (CPR) is crucial during sudden cardiac arrest, and the mortality rate remains markedly high [3]. Despite bystander CPR and early defibrillation improving survival rates, they remain sub-optimal [4,5]. Considering data on CPR duration, the mortality rate is as high as 80–90% for non-shockable rhythms after epinephrine administration for >10 min or shockable rhythms after defibrillation and epinephrine for >20 min [6]. Because CPR only supplies a low-flow status, a CPR duration of >15 min is considered refractory cardiac arrest, and new strategies need to be developed to improve survival [7]. The application of extracorporeal CPR (ECPR) is gaining widespread momentum owing to its capacity for more appropriate blood flow and improvement in ischemia/reperfusion injury after OHCA [8]. In recent years, ECPR has been reported to substantially enhance survival compared with conventional CPR, although the benefit in terms of awakening rate remains poorly clarified [9,10,11,12]. In patients with refractory cardiac arrest, ECPR has been shown to have an improved but still high mortality rate (50–70%) compared to conventional CPR [6].

Levosimendan, an inodilator, increases both heart contractility and vasodilatation, to improve systemic perfusion [13]. Levosimendan has been used in acute cardiac care, including coronary artery bypass graft surgery, advanced heart failure, and rescue therapy for cardiogenic shock [13,14]. Recently, levosimendan was administered during weaning from extracorporeal membrane oxygenation (ECMO) [14,15]. However, the survival benefits associated with the use of levosimendan during ECPR remain unclear. In the current study, we examined the potential effects of levosimendan use on all-cause mortality in patients with OHCA who underwent ECPR.

## 2. Materials and Methods

### 2.1. Study Design and Population

Data were retrieved from electronic hospital records, and written informed consent was waived for this study given its retrospective design. The covariables were age, sex, CPR duration, initial shockable or non-shockable rhythm, previous chronic medication, coronary angiography, mechanical circulatory support (e.g., intra-aortic balloon pump, extracorporeal membrane oxygenation), vasoactive-inotropic score (VIS) at admission, lactate levels, and echocardiography data at 24 h after ECPR. Cardiogenic shock was evaluated by VIS, and lactate levels and cardiac output were measured by echocardiography.

**Figure 1:**
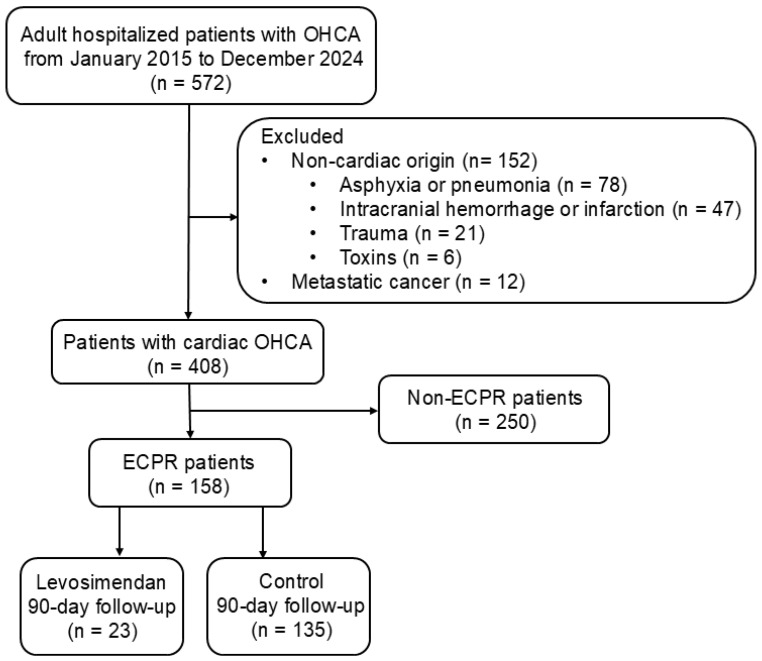
STROBE flow chart for OHCA survivors. A total of 572 adult patients with OHCA, hospitalized between January 2015 and December 2024, were enrolled for further evaluation. Subsequently, 164 patients with OHCA of non-cardiac origin or metastatic cancer were excluded, and 408 eligible patients were analyzed. However, 250 patients did not receive ECMO support. A total of 158 patients who received ECPR underwent further analyses. Additionally, 23 and 135 patients in the levosimendan and control groups, respectively, underwent a 90-day follow-up. ECPR, extracorporeal cardiopulmonary resuscitation; OHCA, out-of-hospital cardiac arrest.

This observational, retrospective cohort study included 158 adult patients with OHCA who underwent ECPR and were consecutively admitted to the intensive care unit of China Medical University Hospital, Taichung, Taiwan, between January 2015 and December 2024. Patients with OHCA of non-cardiac origin, including respiratory distress (e.g., asphyxia, pneumonia), cerebral vascular accident (e.g., intracranial hemorrhage, cerebral infarction), trauma, and toxins were excluded (STROBE flowchart; Figure 1). Patients with metastatic cancer were also excluded for reasons of complexity. The inclusion criteria included adult hospitalized cardiac OHCA (age ≥ 18 years). About 70% of cases were due to coronary heart disease, mainly acute myocardial infarction. The remaining 30% involved heart failure associated with cardiomyopathy or idiopathic ventricular tachyarrhythmia. The study design conformed to the ethical guidelines of the Institutional Review Board of China Medical University Hospital (CMUH112-REC3-016).

### 2.2. Vasopressors (Epinephrine, Norepinephrine, and Vasopressin)

Epinephrine acts on the β1, β2, and α1 adrenergic receptors to promote inotropy and cardiac pumping and induce vasoconstriction. During the first phase of cardiopulmonary resuscitation, high-dose epinephrine is commonly used to facilitate the restoration of cardiac pumping. Norepinephrine acts on α1 and β1 adrenergic receptors. Because of its high affinity for α1 receptors, norepinephrine is typically administered as the first-line vasoconstrictor in a state of shock. Vasopressin acts on the V1 receptor. Owing to its high potency for vasoconstriction, vasopressin is often used as a second-line vasoconstrictor when norepinephrine fails to adequately improve blood pressure.

### 2.3. Inotropes (Dopamine, Dobutamine, Milrinone, and Levosimendan)

Dopamine and dobutamine primarily act on the β1 adrenergic receptor. Dopamine exerts a dose-dependent response. Low-dose dopamine (2–5 µg/kg/min) acts on dopaminergic and β1 adrenergic receptors to enhance renal and interstitial circulation; medium-dose dopamine (5–10 µg/kg/min) acts on β1 adrenergic receptors to increase heart rate and cardiac contractility; and high-dose dopamine (10–20 µg/kg/min) acts on α1 and β1 adrenergic receptors, increasing not only heart rate and cardiac contractility but also blood pressure. Unlike dopamine, dobutamine acts on β1 and β2 adrenergic receptors to increase cardiac contractility and is commonly used in patients with acute heart failure or low cardiac output syndrome. Milrinone is a phosphodiesterase 3 inhibitor that inhibits phosphodiesterase activity, suppressing the hydrolysis of the secondary messenger (cAMP), thereby increasing the cAMP concentration in cells. Milrinone exerts cardiac contractile and vasodilatory effects. Levosimendan is a calcium sensitizer that promotes the binding of myocardial troponin C to calcium, thereby enhancing cardiac contractility. Unlike other inotropes, OR-1855 and OR-1896 maintain cardiac contractility for 1–2 weeks and aid in cardiac recovery after low cardiac output, cardiac arrest, or coronary artery bypass grafting. Levosimendan has been found to exert additional effects on smooth muscles and mitochondria through K_ATP_ channels, thereby dilating blood vessels and protecting the heart.

### 2.4. VIS and SCAI Staging at Admission

The VIS at admission was calculated using the dosing rates of vasoactive and inotropic agents (μg/kg/min or unit/kg/min) administered at admission [16]. The VIS was calculated as follows: (epinephrine [μg/kg/min] × 100) + (norepinephrine [μg/kg/min] × 100) + (vasopressin [unit/kg/min] × 10,000) + (dopamine [μg/kg/min]) + (dobutamine [μg/kg/min]) + (milrinone [µg/kg/min] × 10) [17]. Vasopressors can lead to inadequate perfusion, resulting in increased lactate concentrations. Inotropic agents can induce arrhythmia as a side effect, while inodilators cause hypotension due to vasodilatation. VIS at admission has been used to evaluate the severity of shock. A VIS value of >30 indicated higher severity of shock [18]. All ECPR patients were in cardiogenic shock. The Society for Cardiovascular Angiography and Interventions (SCAI) Stage C is ECPR without hypotension or hypoperfusion, Stage D is ECPR with persistent hypotension or hypoperfusion, and Stage E is refractory OHCA requiring ≥2 vasoactive inotropic agents in addition to epinephrine [19].

### 2.5. Left Ventricular Function Test at 24 h After OHCA

Data on left ventricular ejection fraction (LVEF) 24 h after OHCA were acquired by echocardiography using the Simpson method under an apical 4-chamber view and confirmed by two specialists. LVEF ≤ 40% indicates systolic heart failure. Left ventricular cardiac index (LVCI) data were also acquired using echocardiography via the velocity-time integral of the left ventricular outflow tract. LVCI ≤ 2.2 L/min/M^2^ indicates low cardiac output. Cardiac power output (CPO) is the product of cardiac output and the mean arterial pressure divided by 451; a CPO of <0.6 W indicates cardiogenic shock [20].

### 2.6. Levosimendan Use Within 72 h of ECPR

Levosimendan within 72 h of ECPR was administered at the discretion of the specialist if the systolic blood pressure was >90 mmHg without vasopressor use. Levosimendan was administered as a 12.5 mg dose diluted to 500 mL in 5% dextrose and maintained at 0.05–0.1 μg/kg/min without loading. Following continuous infusion, coadministration of low-dose norepinephrine vasopressors, inotropic agents, or beta-blockers was permitted.

### 2.7. Statistical Analysis

Sample size was determined to detect a clinically significant 35% reduction in the primary endpoint. Considering an ECPR mortality rate of 55% in the control group and mortality rate of 20% in the levosimendan group, 120 patients (100 vs. 20) are required with 85% power at a bilateral alpha risk of 0.05, assuming that 10% of cases would be non-evaluable. Data values are expressed as mean ± standard deviation (SD) or absolute number and percentages. Student’s *t*-test was used to determine *p*-values, with a *p*-value of <0.05 deemed statistically significant. Kaplan–Meier 90-day survival curves were used to compare the levosimendan and control groups. Multivariate Cox logistic regression analysis was performed to identify the independent risk factors for ECPR mortality. The model passed the proportional hazards hypothesis test, and the variance inflation factor of these covariates was set to <10 to avoid multicollinearity. The 90-day hazard ratio, 95% confidence interval (CI), and related significant values obtained from the regression analysis are reported. Statistical significance was set at 5%. All statistical analyses were performed using SPSS version 30.0 (IBM Corp., Armonk, NY, USA).

## 3. Results

### 3.1. Baseline Clinical Characteristics

Table 1 summarizes the baseline clinical characteristics of the study population, which was classified based on levosimendan administration after ECPR. The mean patient age at diagnosis was 54.3 ± 13.9 years, and most were male (87.3%). Primary medical history included hypertension, hyperlipidemia, diabetes mellitus, chronic heart failure, and coronary artery disease as possible risk factors for ECPR.

Despite the high proportion of witnessed cardiac arrest (69.0%), bystander CPR (43.7%), and initial shockable rhythm (75.3%), the no-flow time was 4.8 ± 1.7 min, CPR duration was 34.8 ± 23.9 min, initial arterial pH was 7.01 ± 0.18, and total epinephrine during CPR was 9.3 ± 6.8 mg. The cardiac arrest hospital prognosis (CAHP) score was 151.8 ± 39.4. The leading cause of cardiac OHCA after ECPR was coronary heart disease (67.7%), including left mainstem disease (14.6%) and triple-vessel disease (35.4%). Patients with ST-segment elevation myocardial infarction (42.4%) and non-ST-segment elevation myocardial infarction with hemodynamic instability (20.3%) underwent percutaneous coronary intervention (62.7%). Approximately 31.0% of patients received intra-aortic balloon pumps on ECMO, and 71.5% received targeted temperature management.

The VIS at admission was as high as 33.6 ± 26.2. Echocardiography at 24 h after ECPR revealed that LVEF was 31.1 ± 14.9%, LVCI was 1.62 ± 0.99 L/min/M^2^, and CPO was 0.49 ± 0.33 W. The levosimendan and control groups had similar baseline clinical characteristics.

### 3.2. Follow-Up of Laboratory Tests on the Day of Admission

Table 2 shows the results of laboratory tests at 6 h and 24 h after OHCA. Blood counts were performed on the day of admission. On the first day after ECPR, the white blood cell count increased from 13.9 to 15.3 K/μL, particularly the neutrophil count from 7.8 to 13.1 K/μL. A decrease in hemoglobin from 12.7 to 11.2% and platelet count from 188.3 to 132.9 K/μL was attributed to inflammation-related consumption.

Among the biochemical indices, markers related to heart, liver, and kidney function were significantly elevated following OHCA and ECPR because of organ damage. Although the mean lactate level decreased from 15.7 to 6.9 mmol/L, it remained above the threshold value of 5 mmol/L for critical status. The levosimendan and control groups had similar baseline blood counts and biochemical indices.

### 3.3. Analyses of Sequential Organ Failure Assessment (SOFA), Length of Stay, Clinical Outcomes, and Cerebral Performance Category (CPC)

Table 3 uses the SOFA score including six parameters, namely the heart, lung, brain, liver, kidney, and platelet levels, to assess organ perfusion. The mean SOFA score on day 1 was 14.3. After levosimendan administration, the SOFA score improved, decreasing to 11.6 on day 7. However, the SOFA score increased in the absence of levosimendan administration, reaching 16.2 on day 7. Hospital stay was longer in the levosimendan group than in the control group (38.0 days vs. 24.5 days, *p* < 0.001). The rates of ECMO weaning failure and 90-day mortality were significantly lower in the levosimendan group than in the control group (13.0% vs. 52.6% and 17.4% vs. 57.0%, respectively; all *p* < 0.001).

CPC is used to evaluate neurological outcomes as follows: CPC 1: normal or mild disability; CPC 2: moderate disability; CPC 3: severe disability; CPC 4: vegetative status or coma; CPC 5: brain death. Patients with CPC 1–2 had favorable neurological outcomes, while those assigned CPC 3–5 had poor neurological outcomes. Although the levosimendan group had a lower incidence of poor neurological outcomes than the control group, the difference was statically non-significant (47.8% vs. 71.8%, *p* = 0.06).

### 3.4. Kaplan–Meier Survival Curves of ECPR

**Figure 2:**
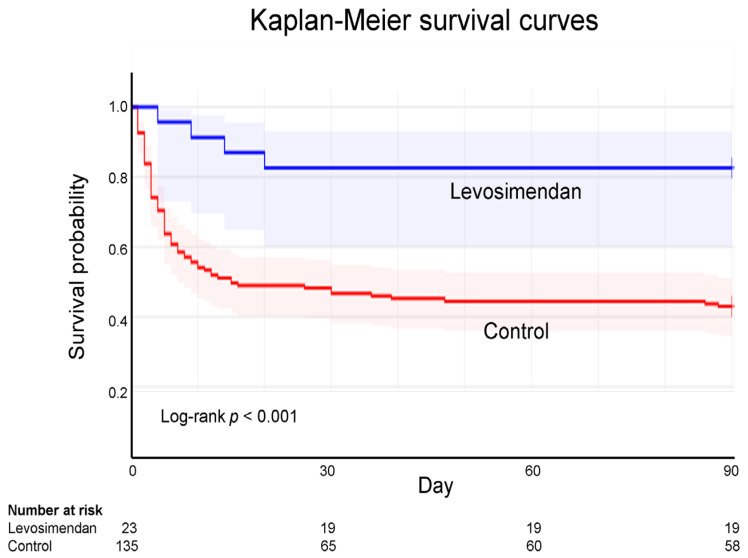
Kaplan–Meier survival curves for extracorporeal cardiopulmonary resuscitation according to levosimendan administration. According to Kaplan–Meier curve analysis, the 90-day survival rate following ECPR is 82.6% and 43.0% for the levosimendan and control groups, respectively.

ECPR analysis was conducted using Kaplan–Meier survival curves. The 90-day follow-up analysis revealed the survival rate was 82.6% in the levosimendan group but only 43.0% in the control group, indicating a significant difference (log-rank *p* < 0.001; Figure 2).

### 3.5. Ninety-Day Mortality Rate-Adjusted Covariables Using Cox Regression Model

**Figure 3:**
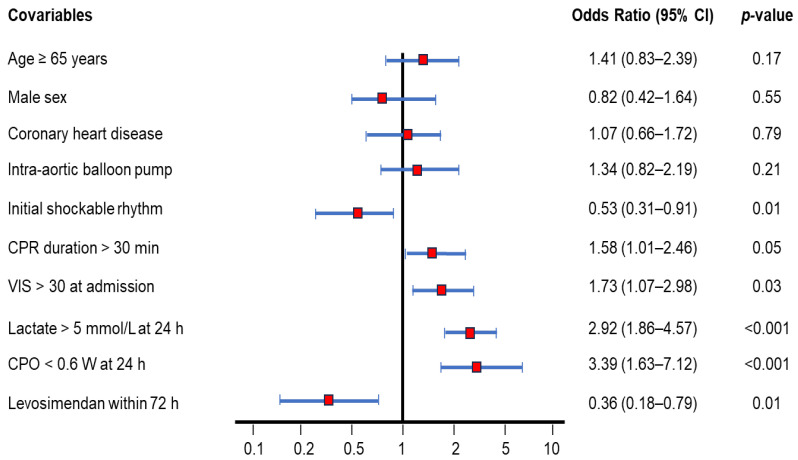
Ninety-day mortality rate-adjusted covariables of OHCA survivors after ECPR using the Cox regression model. Several covariables were analyzed as risk factors for 90-day mortality. Covariables include age ≥ 65 years (odds ratio: 1.41, 95% CI: 0.83–2.39), male sex (odds ratio: 0.82, 95% CI: 0.42–1.64), coronary heart disease (odds ratio: 1.07, 95% CI: 0.66–1.72), add-on intra-aortic balloon pump (odds ratio: 1.34, 95% CI: 0.82–2.19), initial shockable rhythm (odds ratio: 0.53, 95% CI: 0.31–0.91), CPR duration > 30 min (odds ratio: 1.58, 95% CI: 1.01–2.46), VIS > 30 at admission (odds ratio: 1.73, 95% CI: 1.07–2.98), lactate > 5 mmol/L at 24 h (odds ratio: 2.92, 95% CI: 1.86–4.57), CPO < 0.6 W at 24 h (odds ratio: 3.39, 95% CI: 1.63–7.12), and levosimendan administration within 72 h (odds ratio: 0.36, 95% CI: 0.18–0.79). CPO, cardiac power output; CPR, cardiopulmonary resuscitation; ECPR, extracorporeal cardiopulmonary resuscitation; OHCA, out-of-hospital cardiac arrest; VIS, vasoactive-inotropic score.

Figure 3 illustrates several covariates analyzed as risk factors for 90-day mortality after ECPR. Independent hazard factors in descending order were as follows: CPO < 0.6 W (odds ratio: 3.39, 95% CI: 1.64–7.12, *p* < 0.001); lactate > 5 mmol/L (odds ratio: 2.92, 95% CI: 1.86–4.57, *p* < 0.001); VIS at admission > 30 (odds ratio: 1.73, 95% CI: 1.07–2.98, *p* = 0.03); CPR duration > 30 min (odds ratio: 1.58, 95% CI: 1.01–2.46, *p* = 0.05). Age, sex, coronary heart disease, and add-on intra-aortic balloon pump did not significantly impact the 90-day mortality. Importantly, initial shockable rhythm (odds ratio: 0.53, 95% CI: 0.31–0.91, *p* = 0.01) and levosimendan within 72 h (odds ratio: 0.36, 95% CI: 0.18–0.79. *p* = 0.01) were identified as independent protective factors.

## 4. Discussion

In the current study, we evaluated the potential protective factors that could improve all-cause mortality after ECPR in patients with OHCA. In this retrospective study, we found that the use of levosimendan within 72 h yielded a good survival benefit. However, the appropriate timing and conditions for use warrant further investigation.

As an inodilator, levosimendan has been widely used to treat acute and advanced heart failure in Europe and Asia for the past two decades [21,22,23]. Levosimendan, a novel myofilament calcium sensitizer, binds myocardial troponin C with calcium to enhance heart contractility. The circulating metabolites OR-1855 and OR-1896 are formed slowly and maintain heart contractility after 24 h for 1–2 weeks. Levosimendan can also induce smooth muscle relaxation via K_ATP_ activation to enhance vasodilation and protect the heart via mitochondrial K_ATP_ activation [21,22]. Therefore, maximal pulmonary capillary wedge pressure was reduced 6 h after infusion, and maximal cardiac output was enhanced 24 h after infusion [24,25]. Additionally, levosimendan has been employed to address other conditions, including cardiac surgery (especially in coronary artery bypass grafting), right heart failure, tachycardia, postpartum cardiomyopathy, stunned myocardium, calcium channel blocker toxicity, and sepsis, not only benefiting the heart but also providing systemic support [24]. In recent years, levosimendan has been successfully used in cardiogenic shock as an adjunct therapy with vasopressors or antiarrhythmic drugs [25].

Weaning from ECMO can be challenging because of poor heart recovery. Although dopamine inotropy was frequently attempted 10 years ago, the ECMO weaning failure rate was high owing to the adverse effects of ventricular arrhythmias. In recent years, the use of dobutamine has been recommended as the first inotropic agent for ECMO weaning rather than dopamine, given the fewer ventricular arrhythmias and mild vasodilatory effects [26]. Milrinone, an inodilator, has been shown to exert similar results [27]. Levosimendan is the most recent successful inodilator used in ECMO weaning, inducing inotropic and vasodilatory effects similar to milrinone, with a lower myocardial oxygen demand than milrinone or dobutamine [25,27,28]. ECPR combines ECMO and CPR after cardiac arrest. OHCA of cardiac origin is commonly attributed to acute myocardial infarction, heart failure, or idiopathic ventricular arrhythmia. Levosimendan exerts inotropic and vasodilatory effects during ECMO weaning and facilitates cardiac recovery from CPR-related myocardial stunning [15,29,30]. The all-cause mortality rate markedly decreased as the ECMO weaning rate increased [31,32]. The increased perfusion due to levosimendan use also improves cardiac and multiorgan function. Meta-analyses have confirmed the overall decline in all-cause mortality after levosimendan administration [33,34].

Myocardial dysfunction is a key factor governing the response to levosimendan. Because of its inotropic effect, levosimendan improves LVEF, particularly in the stunned myocardium. Moreover, levosimendan improves low cardiac output syndrome owing to its combined inotropic and vasodilatory effects [35]. Follow-up data obtained through echocardiography at 24 h post-ECPR revealed a mean LVEF of 31.2% (<40%), LVCI of 1.73 L/min/m^2^ (<2.2 L/min/m^2^), and CPO of 0.54 W (<0.6 W). Notably, a poor systolic function (LVEF < 25%) can impact prognosis [36]. In this situation, given the lack of viable myocardium, a left ventricular assist device or heart transplantation should be considered in addition to levosimendan [37]. Cardiogenic shock status, composed of hypotension, hypoperfusion, and low cardiac output, obviously affects clinical outcome. Aggravated covariables of ECPR include VIS, lactate levels, and CPO, in addition to CPR duration. VIS represents the severity of hypotension in influencing the clinical outcome; the odds ratio was 1.73, while VIS was > 30 at admission [38]. Lactate levels and status of anaerobic metabolism are indicative of multiorgan poor perfusion. For a lactate level of ˃5 mmol/L 24 h post-ECPR affecting the clinical outcome, the odds ratio was 2.92, consistent with the findings of previous studies [39]. CPO < 0.6 W at 24 h indicates cardiogenic shock, with the highest odds ratio being 3.39. Combining the VIS, lactate levels, and CPO has emerged as a new strategy to predict severity of shock and mortality in critically ill patients [40,41].

In addition to the heart, levosimendan improves perfusion in other organs. Recently, levosimendan was shown to alleviate renal function through renal vasodilation, particularly in patients with cardiorenal syndrome [42]. In our study, the levosimendan group had lower SOFA scores than the control group on days 3, 5, and 7 (13.4 vs. 15.8, *p* = 0.03; 12.7 vs. 16.0, *p* = 0.01; 11.6 vs. 16.2, *p* = 0.003, respectively). This finding could be largely attributed to cardiac and kidney functional recovery. Early administration of levosimendan is crucial because myocardial and renal dysfunction as well as ischemia/reperfusion injury can affect outcomes in the first 72 h. In the current study, the use of levosimendan within 72 h enhanced inotropy and vasodilatation and improved clinical outcomes. Along with supportive ECMO, early levosimendan administration could effectively improve clinical outcomes [43]. Conversely, delaying levosimendan administration for ˃72 h failed to improve ECMO weaning rates or clinical outcomes [44]. The downward trend in the incidence of poor neurological outcomes was statistically non-significant (47.8% vs. 71.8%, *p* = 0.06). This finding implies that, in addition to increased tissue perfusion (e.g., ECMO and levosimendan) and targeted temperature management, other factors must be explored for brain recovery.

This study has some limitations. First, the number of patients who received ECPR with levosimendan administration was relatively small, restricting the confidence level. Second, this study was conducted at a single center and may not be applicable to other hospitals. Third, refractory cardiogenic shock including lactate levels > 5 mmol/L or CPO < 0.6 W at 24 h after ECPR notably diminish the ECMO weaning and bridge-free survival rate [45]. Finally, we need to conduct prospective randomized studies to verify the findings under certain special circumstances.

## 5. Conclusions

In the current study, the levosimendan group had lower rates of ECMO weaning failure and 90-day all-cause mortality than the control group (13.0% vs. 52.6% and 17.4% vs. 57.0%, respectively; both *p* < 0.001). According to the 90-day Kaplan–Meier survival curve analysis, the survival rates for the levosimendan and control groups were 82.6% and 43.0%, respectively (log-rank *p* < 0.001). The hazard ratio for levosimendan administration within 72 h was 0.36 (95% CI: 0.18−0.79, *p* = 0.01). Based on these findings, levosimendan should be included as a protective agent for patients undergoing ECPR after OHCA.

## Figures and Tables

**Table 1:** Baseline clinical characteristics of study subjects depending on whether levosimendan was administered after ECPR.

		Levosimendan (*n* = 23)	Control (*n* = 135)	*p*-Value
**Age (y), mean ± SD**		54.1 ± 12.9	54.3 ± 14.1	0.96
**Male sex, *n* (%)**		21 (91.3)	117 (86.7)	0.54
**BMI (kg/m^2^), mean ± SD**		27.7 ± 4.1	26.8 ± 4.8	0.34
**Medical history,** ***n* (%)**	Hypertension	13 (56.5)	52 (38.5)	0.11
	Hyperlipidemia	9 (39.1)	49 (36.3)	0.66
	Diabetes mellitus	5 (21.7)	28 (20.7)	0.91
	Chronic heart failure	4 (17.4)	20 (14.8)	0.75
	Coronary artery disease	4 (17.4)	26 (19.3)	0.83
	End-stage renal disease	1 (4.3)	7 (5.2)	0.87
	Cerebrovascular disease	1 (4.3)	3 (2.2)	0.55
	Chronic obstructive pulmonary disease	1 (4.3)	1 (0.7)	0.15
**Witnessed cardiac arrest, *n* (%)**		17 (74.3)	92 (68.2)	0.48
**Bystander CPR, *n* (%)**		12 (52.2)	57 (42.2)	0.29
**Initial shockable rhythm, *n* (%)**		2 (91.3)	98 (72.6)	0.05
**No-flow time (min), mean ± SD**		4.6 ± 1.5	4.9 ± 0.4	0.51
**CPR duration (min), mean ± SD**		30.7 ± 13.7	35.5 ± 25.3	0.37
**Initial arterial pH,** **mean ± SD**		7.04 ± 0.16	7.01 ± 0.19	0.39
**Total Epinephrine while CPR (mg), mean ± SD**		9.3 ± 5.7	9.3 ± 7.1	0.98
**CAHP score, mean ± SD**		140.3 ± 26.7	153.8 ± 7.1	0.13
**CHD,** ***n* (%)**	Coronary angiography	15 (65.2)	92 (68.1)	0.78
	Left main disease	6 (26.1)	17 (12.6)	0.09
	Triple-vessel disease	9 (39.1)	47 (34.8)	0.69
**AMI,** ***n* (%)**	Percutaneous coronary intervention	14 (60.9)	85 (63.0)	0.85
	ST-elevation myocardial infarction	12 (52.2)	55 (40.7)	0.31
**Intra-aortic balloon pump, *n* (%)**		11 (47.8)	38 (28.1)	0.06
**Targeted temperature management, *n* (%)**		17 (73.9)	96 (71.1)	0.78
**Cardiogenic shock, *n* (%)**	SCAI Stage C	3 (13.0)	24 (17.8)	0.58
	SCAI Stage D	9 (39.1)	50 (37.0)	0.85
	SCAI Stage E	11 (47.8)	61 (45.2)	0.82
**VIS** **at admission, mean ± SD**		34.9 ± 23.6	32.6 ± 27.6	0.72
Norepinephrine (μg/kg/min), mean ± SD		0.3 ± 0.2	0.2 ± 0.2	
Dopamine (μg/kg/min), mean ± SD		6.3 ± 7.7	8.1 ± 9.3	
Dobutamine (μg/kg/min), mean ± SD		0.5 ± 1.5	0.2 ± 0.9	
**LVEF at 24 h (%), mean ± SD**		31.2 ± 12.2	31.0 ± 15.4	0.97
**LVCI at 24 h (L/min/M^2^), mean ± SD**		1.73 ± 0.83	1.60 ± 1.02	0.56
**CPO at 24 h (W), mean ± SD**		0.54 ± 0.26	0.48 ± 0.35	0.41

AMI, acute myocardial infarction; BMI, body mass index; CAHP, cardiac arrest hospital prognosis; CHD, coronary heart disease; CPO, cardiac power output; CPR, cardiopulmonary resuscitation; ECPR, extracorporeal cardiopulmonary resuscitation; LVCI, left ventricular cardiac index; LVEF, left ventricular ejection fraction; SCAI, society for cardiovascular angiography and interventions; SD, standard deviation; VIS, vasoactive-inotropic score.

**Table 2:** Baseline blood counts and biochemical indices of study subjects at 6 and 24 h depending on whether levosimendan was administered after ECPR.

	Levosimendan (*n* = 23)	Control (*n* = 135)	*p*-Value
**Blood counts**			
**WBC (K/μL) at 6 h, mean ± SD**	14.0 ± 6.8	13.9 ± 6.0	0.98
**WBC (K/μL) at 24 h, mean ± SD**	14.4 ± 7.8	15.5 ± 7.0	0.51
**Neutrophils (K/μL) at 6 h, mean ± SD**	7.6 ± 5.8	7.9 ± 5.0	0.78
**Neutrophils (K/μL) at 24 h, mean ± SD**	12.4 ± 7.4	13.2 ± 6.5	0.57
**Hemoglobin (%) at 6 h, mean ± SD**	13.2 ± 2.4	12.5 ± 2.6	0.05
**Hemoglobin (%) at 24 h, mean ± SD**	12.1 ± 1.9	11.0 ± 2.5	0.06
**Platelet (K/μL) at 6 h, mean ± SD**	182.5 ± 74.8	189.3 ± 140.8	0.82
**Platelet (K/μL) at 24 h, mean ± SD**	123.5 ± 53.3	134.6 ± 72.0	0.48
**Biochemical indices**			
**Troponin-I (ng/mL) at 6 h, mean ± SD**	6.9 ± 5.7	4.3 ± 3.8	0.08
**Troponin-I (ng/mL) at 24 h, mean ± SD**	110.2 ± 100.3	80.1 ± 80.8	0.11
**AST (U/L) at 6 h, mean ± SD**	249.6 ± 198.8	305.4 ± 311.4	0.46
**AST (U/L) at 24 h, mean ± SD**	577.6 ± 287.7	812.6 ± 1872.8	0.58
**ALT (U/L) at 6 h, mean ± SD**	125.2 ± 105.4	150.8 ± 177.6	0.53
**ALT (U/L) at 24 h, mean ± SD**	232.6 ± 180.3	272.7 ± 566.0	0.74
**BUN (mg/dL) at 6 h, mean ± SD**	21.0 ± 7.7	24.8 ± 20.6	0.41
**BUN (mg/dL) at 24 h, mean ± SD**	26.2 ± 6.1	28.9 ± 17.6	0.47
**Creatinine (mg/dL) at 6 h, mean ± SD**	1.5 ± 0.4	2.0 ± 2.0	0.22
**Creatinine (mg/dL) at 24 h, mean ± SD**	1.7 ± 0.4	2.0 ± 1.5	0.29
**Lactate (mmol/L) at 6 h, mean ± SD**	14.0 ± 6.3	16.0 ± 6.8	0.21
**Lactate (mmol/L) at 24 h, mean ± SD**	5.1 ± 2.8	7.2 ± 6.2	0.12

AST, aspartate aminotransferase; ALT, alanine aminotransferase; BUN, blood urea nitrogen; ECPR, extracorporeal cardiopulmonary resuscitation; WBC, white blood cells.

**Table 3:** Sub-analyses of SOFA, length of stay, clinical outcomes, and CPC for the study population based on whether levosimendan was administered after ECPR.

	Levosimendan (*n* = 23)	Control (*n* = 135)	*p*-Value
**SOFA on day 1, mean ± SD**	14.1 ± 1.9	14.4 ± 2.5	0.64
**SOFA on day 3, mean ± SD**	13.4 ± 2.6	15.8 ± 5.2	0.03
**SOFA on day 5, mean ± SD**	12.7 ± 3.3	16.0 ± 6.2	0.01
**SOFA on day 7, mean ± SD**	11.6 ± 3.4	16.2 ± 7.0	0.003
**Hospital stay (days), mean ± SD**	38.0 ± 21.7	24.5 ± 26.0	0.02
**ECMO weaning failure, *n* (%)**	3 (13.0)	71 (52.6)	<0.001
**90-day mortality, *n* (%)**	4 (17.4)	77 (57.0)	<0.001
**90-day poor neurological outcomes, *n* (%)**	11 (47.8)	97 (71.8)	0.06
**CPC sub-analyses at 90-day follow-up**			
**CPC 1, *n* (%)**	6 (26.1)	27 (20.0)	
**CPC 2, *n* (%)**	6 (26.1)	11 (8.1)	
**CPC 3, *n* (%)**	5 (21.7)	7 (5.2)	
**CPC 4, *n* (%)**	2 (8.7)	13 (9.6)	
**CPC 5, *n* (%)**	4 (17.4)	77 (57.0)	

CPC, Cerebral Performance Category; ECMO, extracorporeal membrane oxygenation; ECPR, extracorporeal cardiopulmonary resuscitation; SD, standard deviation; SOFA, Sequential Organ Failure Assessment.

## Data Availability

The original contributions presented in this study are included in the article, and further inquiries can be directed to the corresponding authors.

## Abbreviations

CAHP	Cardiac arrest hospital prognosis
CI	Confidence interval
CPC	Cerebral performance category
CPO	Cardiac power output
CPR	Cardiopulmonary resuscitation
ECPR	Extracorporeal cardiopulmonary resuscitation
ECMO	Extracorporeal membrane oxygenation
LVEF	Left ventricular ejection fraction
LVCI	Left ventricular cardiac index
OHCA	Out-of-hospital cardiac arrest
SCAI	Society for cardiovascular angiography and interventions
SOFA	Sequential organ failure assessment
VIS	Vasoactive-inotropic score
